# Strong Selection of a Few Dominant CD8 Clones in a TLR7-Dependent Autoimmune Mouse Model

**DOI:** 10.4049/immunohorizons.1800082

**Published:** 2019-02-11

**Authors:** Peter A. Morawski, Silvia Bolland

**Affiliations:** Laboratory of Immunogenetics, Division of Intramural Research, National Institute of Allergy and Infectious Diseases, National Institutes of Health, Rockville, MD 20852

## Abstract

Systemic lupus is characterized by the expansion of a self-reactive repertoire of B cells and CD4 cells that together promote IgG Ab production against common nuclear Ags. Although several studies have suggested roles for CD8^+^ T cells in lupus, the full contribution of these lymphocytes to disease remains undefined. In particular, few studies have examined TCR clonotypes of the CD8 pool in lupus. We previously described activated but nonpathogenic CD8^+^ T cells in a mouse model of systemic autoimmune disease triggered by increased copy number of the *tlr7* gene (TLR7tg mice), in which some of these T cells accumulate in the brain. In this article, we report, through the analysis of TCRβ sequences, that CD8 cells from TLR7tg animals are strongly selected for a small number of clones, some of them reaching 30% of the repertoire, compared with less than 0.4% for the top clone in any wild type mice. High frequency clones are variable in sequence among individual TLR7tg mice and are distinct from top clones in the control animals, whereas CDR3 sequences of spleen and brain-resident T cells from the same TLR7tg animals have perfect concordance. These results suggest that top CD8 clones are selected in stochastic fashion in each animal but limit further diversification, and that brain-infiltrating CD8 cells in TLR7tg mice are not selected by a common tissue Ag. This kind of extreme clonal dominance and narrowing of the CD8^+^ repertoire might impair anti-viral responses and should be considered as an additional detrimental feature of chronic autoimmune disease.

## INTRODUCTION

A substantial component of systemic autoimmune disease such as lupus involves the breakdown of lymphocyte tolerance, primarily through the expansion of autoreactive CD4^+^ T cells that provide help to B cells, which can in turn produce autoantibodies with various specificities (1). Although comparatively less is understood about how CD8^+^ T cells contribute to disease progression, perturbations in CTL function are evident in lupus patients (2, 3). Several groups, including ours, have now shown that a functionally competent CD8^+^ T cell compartment is required to restrict disease severity in lupus-prone mice (4–7), and subsets of CD8^+^ T cells have been identified that can suppress excessive CD4^+^ T follicular helper cell help to Bcells, which is essential for maintenance ofself tolerance (8).

We have studied lupus using several disease models including TLR7tg mice. These animals have a severe phenotype that includes glomerulonephritis, anemia, and a lymphoproliferative disorder that mainly impacts the CD4^+^ T and B cell pools, whereas the effect on the CD8 compartment is comparatively minimal (9, 10). We recently showed that the CD8^+^ T cells in these mice are nonpathogenic and have a strong effector phenotype compared with wild type controls, with the most activated cells accumulating in the brain (11). Because of the vast number of potential self-antigens available in both the periphery and brain, an understanding of the Ag specificity of these lymphocytes could help to better understand their relevance and function.

Membrane-bound Ag receptors are required by all lymphocytes for the recognition of peptide fragments bound to and presented by MHC proteins. The TCR serves as the specific Ag receptor on T lymphocytes and is composed of an α and a β-chain (12). The rearrangement of germline encoded variable (V), diversity (D), and joining (J) genes provides the immunological basis for the vast number of possible unique Ag receptors on lymphocytes. In naive mice (13, 14) and in healthy humans (15, 16), certain common patterns of Vβ gene usage can be found in the formation of the TCR repertoire. Previous studies using TCR spectratyping and sequencing of T cells isolated from the blood and kidney of lupus patients revealed deviations from common TCR β-chain usage and alterations in expected CDR3 profiles (17, 18) and identified clonally expanded CD8^+^ T cells associated with renal disease (19). Studies using next-generation TCR sequencing have now identified the presence of public sequences among lupus patients, which could serve as potential biomarkers for disease (20). Although these data collectively demonstrate that the TCR repertoire, and thus, Ag specificity, in lupus is important, we still understand little about the selective induction of autoreactive responses in systemic autoimmunity.

In the current study, we address whether the nonpathogenic peripheral and brain-resident CD8^+^ T cell populations in the TLR7tg model of lupus disease are selected by their Ag specificity. We were interested to understand whether these lupus-prone CD8^+^ T cells respond to environmental factors in a polyclonal fashion or if some strong selective pressures might exist that induce a more Ag-specific response. To accomplish this, we sequenced the TCR β-chains of this population of CD8^+^ T cells. Our results demonstrate strong clonal dominance of CD8^+^ T cells can arise in the context of systemic autoimmune disease, suggesting that the function of these cells might be compromised in some severe cases of disease.

## MATERIALS AND METHODS

### Mice and experimental protocols

The generation of TLR7tg mice has been described previously (9). All experiments in this study used the transgenic line 7.1, which harbors 8- to 16-fold TLR7 gene expression above wild type controls. All mice were maintained on a C57BL/6J background. Male mice between 12 and 13 wk of age were used for these experiments. All animals were housed and studied in accordance with the approved National Institutes of Health Animal Study Protocol, and all experimental protocols were approved by and performed according to National Institutes of Health Animal Care and Use Committee guidelines. All efforts were made to minimize animal suffering and to reduce the number of animals used.

### Vascular labeling of leukocytes

Discrimination of cells in the vasculature versus those in the brain parenchyma was performed as described (21, 22). Briefly, 5 mg of BV421-CD45.2 (1D4; BioLegend) diluted in sterile 1× PBS was injected i.v. once per mouse. Abs were allowed to circulate for a maximum of 3 min, and then mice were euthanized and organs were extracted. Subsequent ex vivo colabeling of tissue-extracted lymphocytes with allophycocyanin/Cy7-CD45.2 (1D4; BioLegend) is done as described below and allows discrimination between vessel (dual CD45.2 labeled) and parenchymal (ex vivo fluorophore only) cells.

### Lymphocyte extraction from tissue

Cell extraction from tissues was modified according to the described protocol (23). For lymphocyte extraction, organs were digested for 30 min at 37°C and shaken at 240 rpm in HBSS containing 1 mg/ml Collagenase Type I (Life Technologies). Digested mix was pelleted, washed, and passed through a 70-μM mesh filter, then resuspended in Percoll (GE Healthcare) diluted to 90% using HBSS. Layers of 60 and 37% Percoll were then sequentially overlayed on the 90% cell–Percoll mix. Cell separation was accomplished by centrifugation at 8°C, 500 × *g* for 18 min without brakes. Cells were washed and resuspended in staining buffer containing 5% FBS in HBBS in preparation for flow cytometry.

### Lymphocyte enrichment and flow cytometric sorting

**FIGURE 1.**
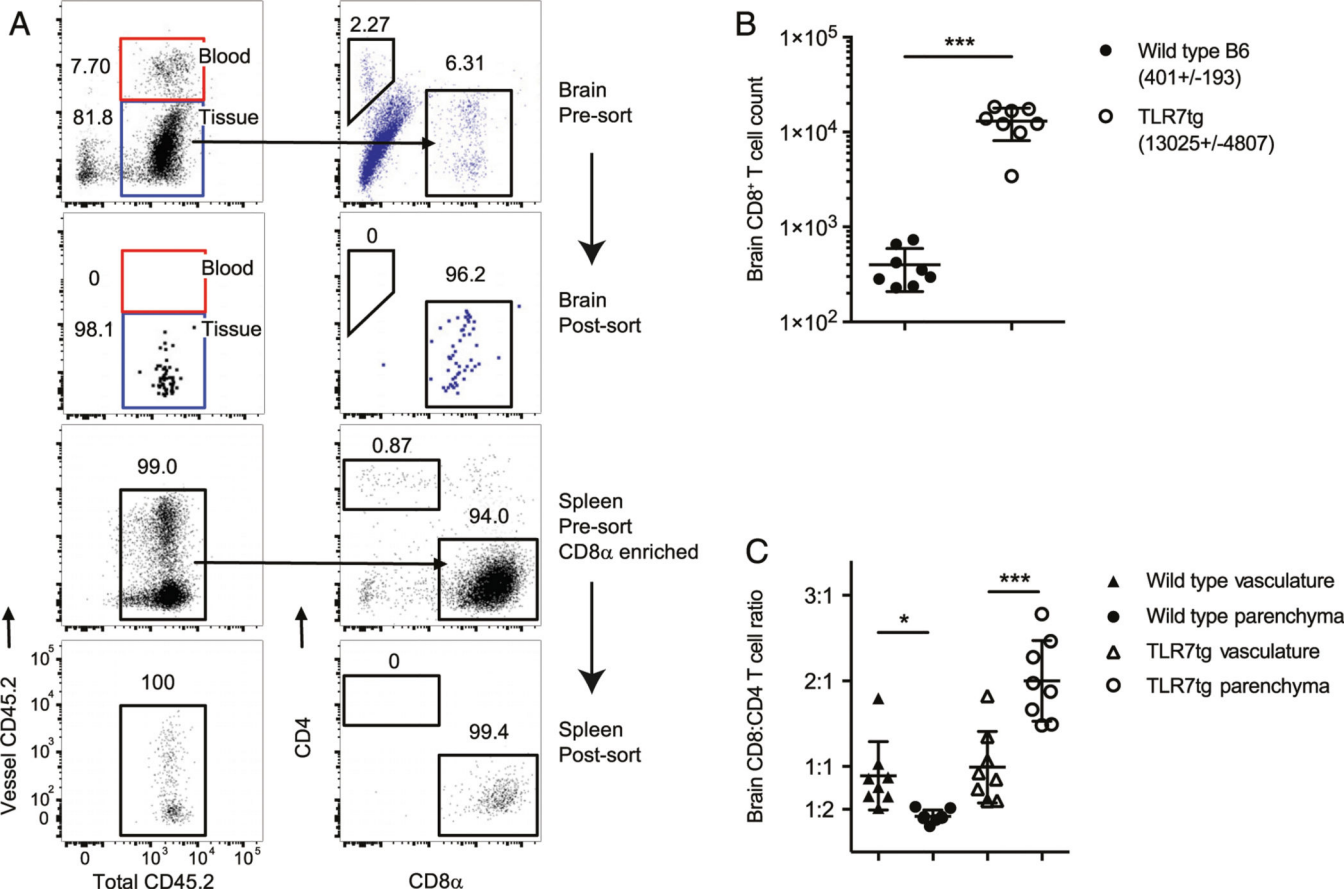
Isolating CD8^+^ T cells from lupus-prone mice. (**A**) Gating strategy (presort) and results (postsort) for flow cytometric isolation of CD8^+^ T cells from wild type B6 and TLR7tg brain and spleen. Discrimination of cells in the parenchyma (tissue) from those in the vasculature (blood) was performed only for brain lymphocytes. Splenic lymphocytes were first positively pre-enriched for CD8α. (**B**) Absolute count of brain CD8^+^ T cells of animals from (A) with mean and SD listed. (C) CD8^+^/CD4^+^ T cell ratio in the brain of animals from (A). Error bars indicate mean + SD. Student *t* test, unpaired (B) or paired (**C**). *p ≤ 0.05, ***p ≤ 0.001. clonality as previously described (26). Error bars indicate mean + SD; *p ≤ 0.05, **p ≤ 0.01, ***p ≤ 0.001, and ****p ≤ 0.0001 (Student *t* test).

Single-cell lymphocyte solutions were prepared from organs as indicated and resuspended in staining buffer containing 5% FBS in HBBS. Pre-enrichment of splenic lymphocytes was performed using PE-labeled CD8^+^ T cell magnetic bead positive selection according to the manufacturer instructions (RoboSep, STEMCELL Technologies). Splenic and brain-resident lymphocytes were identified using mAbs against the following Ags, all from BioLegend unless otherwise noted: PE-CD8α (MAR1), FITC-CD4 (RM4–5), BV711-CD11b (M1/70), BV421-CD45.2 (1D4), allophycocyanin/Cy7-CD45.2 (1D4), and allophycocyanin-TCRβ, (H57–597; eBio science). Samples were sorted on an FACSAria III SORP (BD Biosciences) equipped with violet (403 nm, 100 mW), blue (488 nm, 100 mW), yellow-green (561 nm, 50 mW), and red (638 nm, 150 mW) lasers, then analyzed with FlowJo (Tree Star). Pre- and postsort purity of CD8^+^ T cells are shown in Fig. 1A.

### DNA extraction, TCRβ gene sequencing, and analysis

Frozen cell pellets of sorted wild type B6 and TLR7tg spleen (>2 × 10^5^) and TLR7tg brain (>1 × 10^4^) CD8^+^ T cells were sent on dry ice to Adaptive Biotechnologies (Seattle, WA) for genomic DNA extraction and subsequent TCRβ gene sequencing. Amplification and sequencing of TCRβ CDR3 was performed using the Adaptive Biotechnologies ImmunoSEQ Platform, which combines multiplex PCR with high throughput sequencing and a sophisticated bioinformatics pipeline for TCRβ CDR3 analysis (24, 25). Data analysis was performed using ImmunoSeq Analyzer 3.0 software, also provided by Adaptive Biotechnologies.

### Statistical analysis

Statistical significance of data were calculated with Prism 6.0 software (GraphPad). For comparisons between two normally distributed groups, a two-tailed unpaired *t* test with Welch correction was used. For comparison between more than two groups, statistical analysis was performed using a one-way ANOVA with the Tukey method. Gaussian distribution analysis of CDR3 sequences was used to characterize population

## RESULTS

### The CD8^+^ T cell repertoire in TLR7tg mice is oligoclonal

We previously described the presence of activated nonpathogenic CD8^+^ T cells in the spleen and brain of TLR7tg mice, which we hypothesized could regulate aspects of the systemic disease developed in this model (7). To gain insight into the clonality of these cells, we performed TCRβ sequence analysis of CD8^+^ lymphocytes extracted from the spleens and brains of TLR7tg mice or from spleens of wild type controls. CD8^+^ bead enrichment followed by flow cytometric sorting was used to select for CD8^+^TCRβ^+^CD4^−^CD11b^−^ T cells with a final purity of at least 99% (Fig. 1A).

**FIGURE 2.**
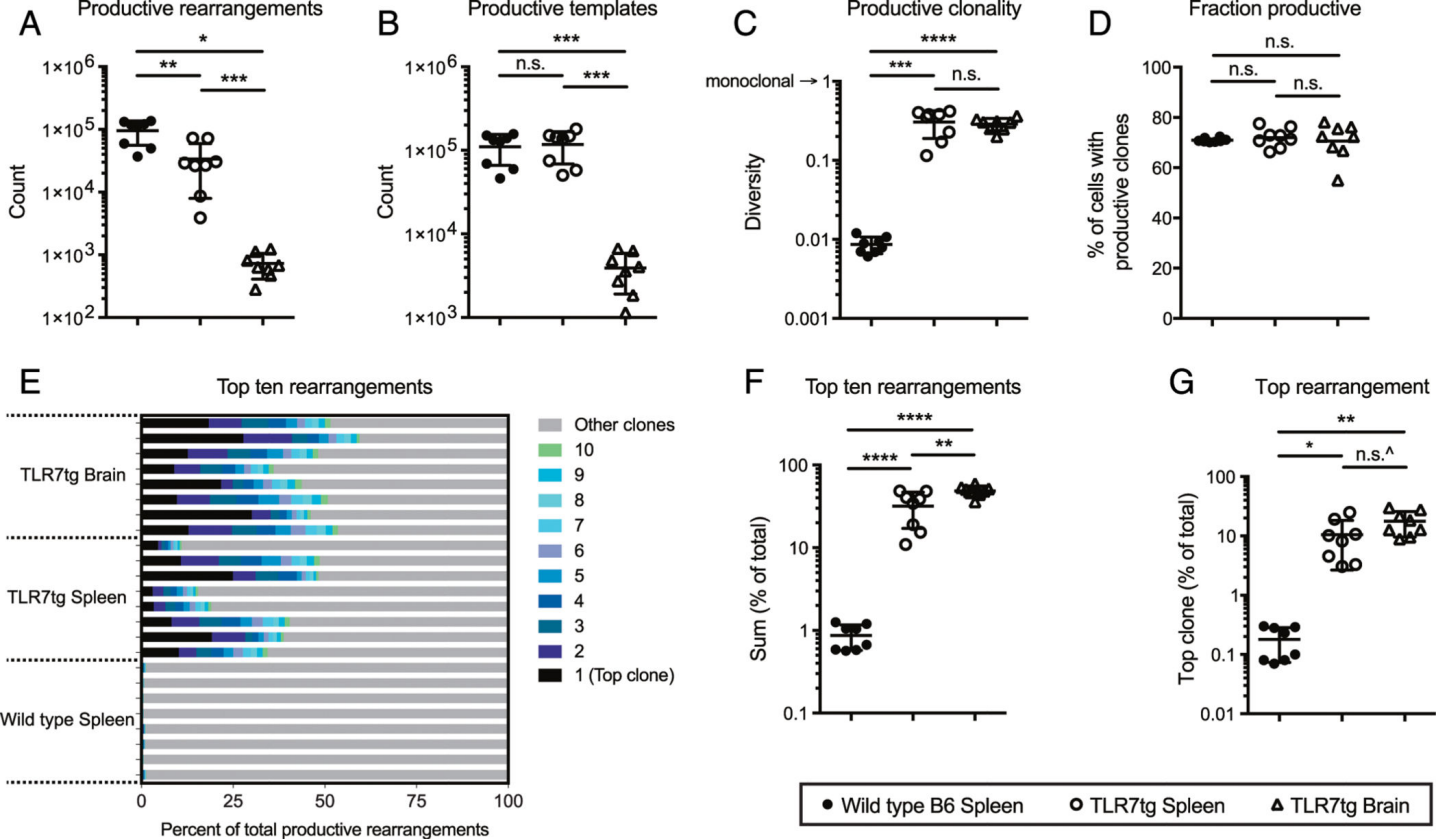
CD8^+^ T cells from lupus-prone mice demonstrate substantial oligoclonality. (**A–D**) Overview of total TCRβ rearrangements from wild type B6 and TLR7tg CD8^+^ T cells from Fig. 1. (A) The count of unique rearrangements in each sample that are in-frame and do not contain a stop codon. (B) The sum of templates for all productive rearrangements in the sample. (C) Clonality measure for each sample calculated over all productive rearrangements. (D) The fraction of productive in-frame templates among all templates. (**E–G**) The frequency (E) and sum (F) of the top 10 clones and the top clone (G) for each sample relative to all rearrangements. Error bars indicate mean + SD. ^*p* = 0.08, *p ≤ 0.05, **p ≤ 0.01, ***p ≤ 0.001, ***p ≤ 0.0001, by Student *t* test.

Brain parenchymal CD8^+^ T cells were distinguished and separated from those in the vasculature using a combination of in vivo intravascular labeling and ex vivo staining as previously described (21, 22). A precise cell count provided during FACS isolation indicated an average of 13,025 CD8^+^ T cells per brain of each TLR7tg mouse compared with an average of only 401 in each wild type control brain (Fig. 1B). Because we required a minimum of 1000 cells for effective TCRβ sequencing analysis, we did not include the wild type brain CD8^+^ T cells in our study. Consistent with our previous findings (7), CD8^+^ T cells outnumbered CD4^+^ T cells in the brain parenchyma by 1.5- to 3-fold, a unique skewing not present in the vasculature or in other organs of lupus-prone mice (Fig. 1C). Genomic DNA from isolated CD8^+^ cells was used for TCRβ sequencing with the Adaptive Biotechnologies ImmunoSEQ Platform. The number of unique in-frame TCR rearrangements per sample in TLR7tg spleens was decreased compared with wild type spleens (Fig. 2A) despite the total cell input (~2 × 10^5^) and the sum of all productive templates being the same in both groups (Fig. 2B). These data can be collectively summarized using a metric to measure total sample diversity, indicating that the peripheral TLR7tg CD8^+^ T cell repertoire has an increased degree of oligoclonality with respect to wild type controls (Fig. 2C). Similar oligoclonality was found in the brain-resident CD8^+^ T cell pool (Fig. 2A–C), indicating that as a population, these cells are largely derived from the same parents clones as those in the periphery. Additionally, an equivalent percentage of cells sequenced from each of the three groups produced in-frame productive rearrangements without a stop codon (Fig. 2D).

An analysis of the most abundant 10 clones in wild type controls reveals an average summative frequency per mouse of <1%, indicative of a polyclonal response (Fig. 2E, 2F). By comparison, the strong oligoclonality of both splenic and brain CD8^+^ T cells from TLR7tg mice can also be seen in the summative frequency of the top 10 clones in each animal, which can amount to 60.5% of the repertoire (Fig. 2E, 2F). In this case, the sum of the top clones in the TLR7tg brain with respect to the entire repertoire was statistically higher relative to the sum of the top clones in the TLR7tg spleen (Fig. 2F). Remarkably, the top unique rearrangement in the total TLR7tg CD8^+^ T cell pool was 30.1% of all productive templates, whereas this number never exceeded 0.4% for the wild type group (Fig. 2G).

### Skewed CDR3 length and VDJβ gene usage in TLR7tg CD8^+^ T cells compared with wild type

**FIGURE 3.**
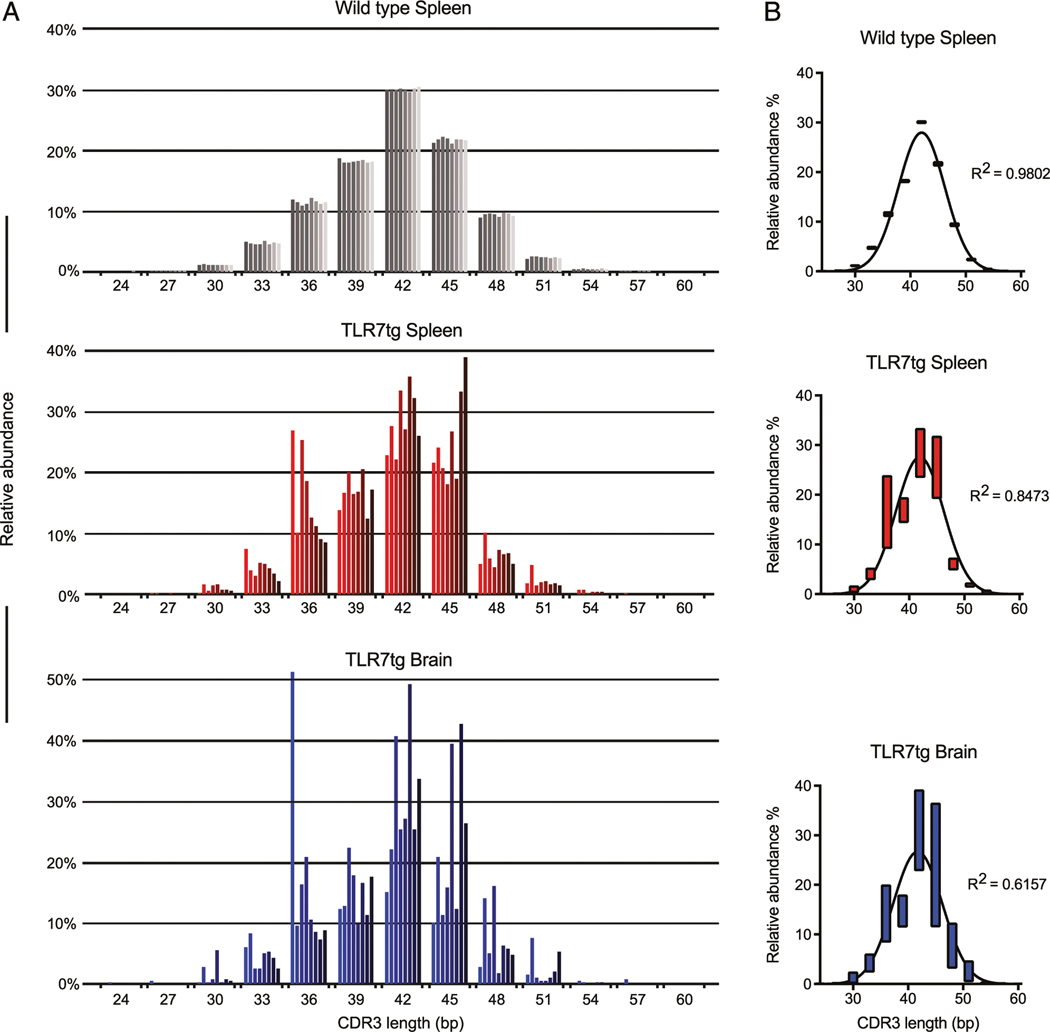
Variable CDR3 lengths support the notion of oligoclonality in CD8^+^ T cells of lupus-prone mice. (**A**) Relative abundance and distribution of CDR3 nucleotide sequence lengths (bp) from purified CD8^+^ T cells of wild type B6 spleen (shades of gray), TLR7tg spleen (shades of red), and TLR7tg brain (shades of blue). Each shaded bar represents a separate sample of the indicated genotype and tissue. For each possible CDR3 length, the order of the eight bars from left to right is matched for spleen and brain samples from TLR7tg mice. (**B**) Average distribution analysis of compiled samples (*n* = 8) from (A). Bars indicate minimum and maximum values centered on the mean. R^2^ values indicate the fit of each curve to normal Gaussian distribution.

An established method of assessing the relative clonality in a given lymphocyte repertoire is through an analysis of the CDR3 lengths within the population. Normal Gaussian distribution of the CDR3 lengths is indicative of polyclonal responses (26). Consistent with this, we found that CDR3 sequence lengths of CD8^+^ T cells from healthy control mice demonstrated a very strong fit to a Gaussian curve centered on a length of 14 aa (Fig. 3A, 3B, top), which is consistent with a recent comprehensive analysis including B6 control animals (27). By comparison, the CDR3 segment lengths of TLR7tg splenic CD8^+^ T cells had a substantial divergence from expected Gaussian distribution (Fig. 3A, 3B, middle). Additional variance was found in brain-isolated cells (Fig. 3A, 3B, bottom). Importantly, despite a narrowing repertoire, the CDR3 sequence distribution across all TLR7tg samples tested is not uniformly directed toward a specific set of rearrangement processes; each of the eight lupus-prone animals exhibit a uniquely skewed abundance of CDR3 lengths. There is, however, a similar pattern of CDR3 length usage between shared spleen and brain samples within each of the same TLR7tg animals (Fig. 3A, 3B, middle and bottom, sequentially matched bars left to right).

**FIGURE 4.**
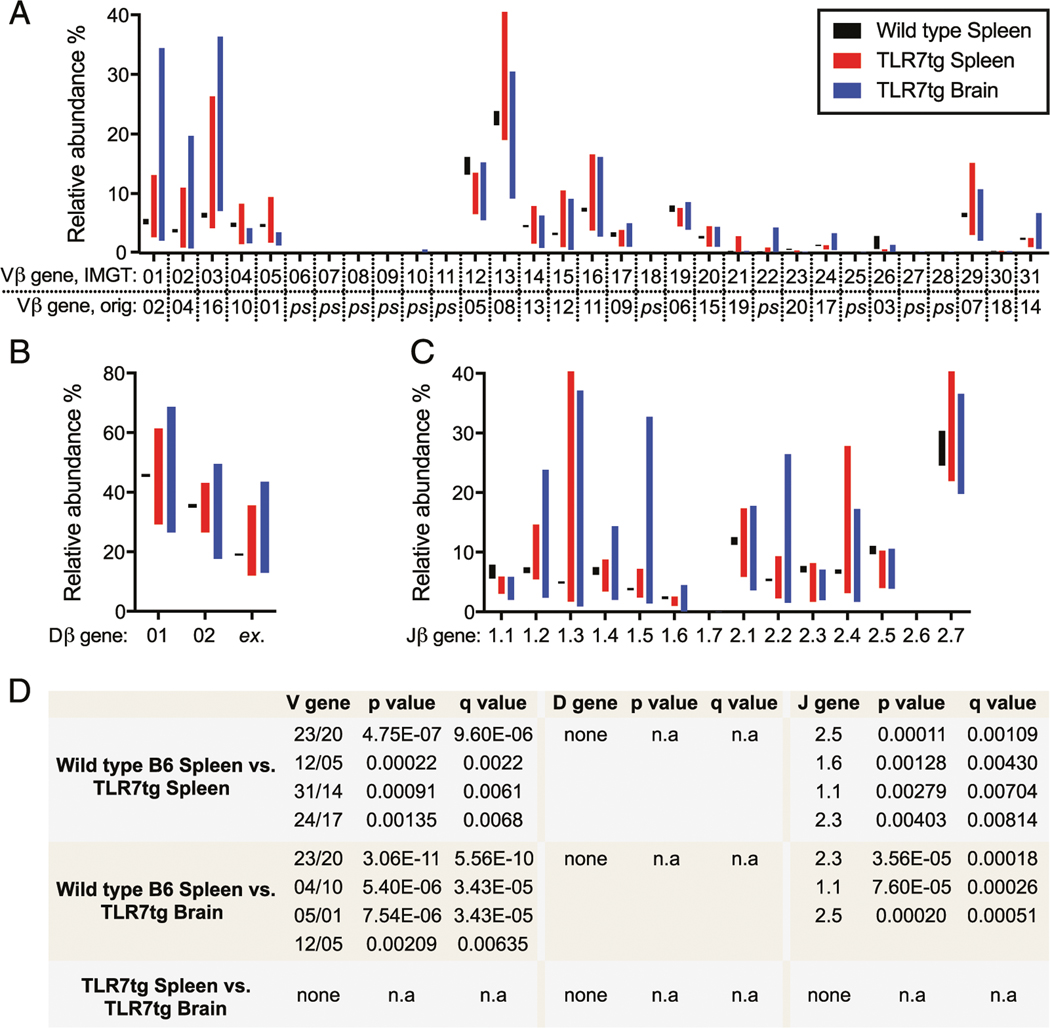
Highly variable VDJβ gene usage in lupus-prone CD8^+^ T cells. (**A**–**C**) Vβ gene (A) usage for CD8^+^ T cells from wild type B6 spleen and TLR7tg spleen and brain. Equivalent IMGT and original (orig.) nomenclature for Vβ genes included. ps, pseudogene. (B) Dβ gene and (C) Jβ gene usage shown as in (A). Bars indicate minimum and maximum values centered on the mean. (**D**) Statistical analysis of discovered significant differences between indicated groups for (A–C). Multiple *t* test grouped analysis used to generate *p* values and false discovery rate–adjusted q values. These results are presented with two forms of nomenclature: the comprehensive international IMGT, and the original, still commonly used nomenclature.

We next analyzed VDJβ gene usage in CD8^+^ T cells from wild type and TLR7tg mice. The results in Fig. 4 include total Vβ gene segment usage and are presented with two forms of nomenclature. The international ImMunoGeneTics information system (IMGT) is the current standard used since 2000 and includes all pseudogenes in the Vβ gene region (28), although many publications and commercial Ab suppliers still adhere to the original distinctions (29). Naive animals rely on a relatively small pool of TCRβ genes in the makeup of their peripheral repertoire (30). Our analysis of the VDJβ gene makeup of the CD8^+^ T cells in wild type B6 mice was consistent with these published results, with the majority of cells using Vβ12 and Vβ13 (Fig. 4A), Dβ1 (Fig. 4B), and Jβ2.7 (Fig. 4C) gene segments. In contrast, cells from the spleen of TLR7tg mice showed highly variable VDJβ usage (Fig. 4A–C) and significant divergence from common gene segments such as Vβ12 and Jβ 2.5 (Fig. 4D). The brain CD8^+^ T cells from TLR7tg mice also used statistically less Vβ12 and Jβ 2.5. In place of these common Vβ and Jβ genes, both the spleen and brain cells from lupus-prone mice used other Vβ and Jβ genes, although no consistent pattern of usage emerged across those samples. In fact, substantial mouse-to-mouse variability of VDJβ genes identified was evident in both TLR7tg splenic and brain CD8^+^ T cell populations (Fig. 4D).

### The peripheral and brain-infiltrating CD8^+^ T cell pools within the same lupus-prone mouse show substantial CDR3 nucleotide sequence sharing

**FIGURE 5.**
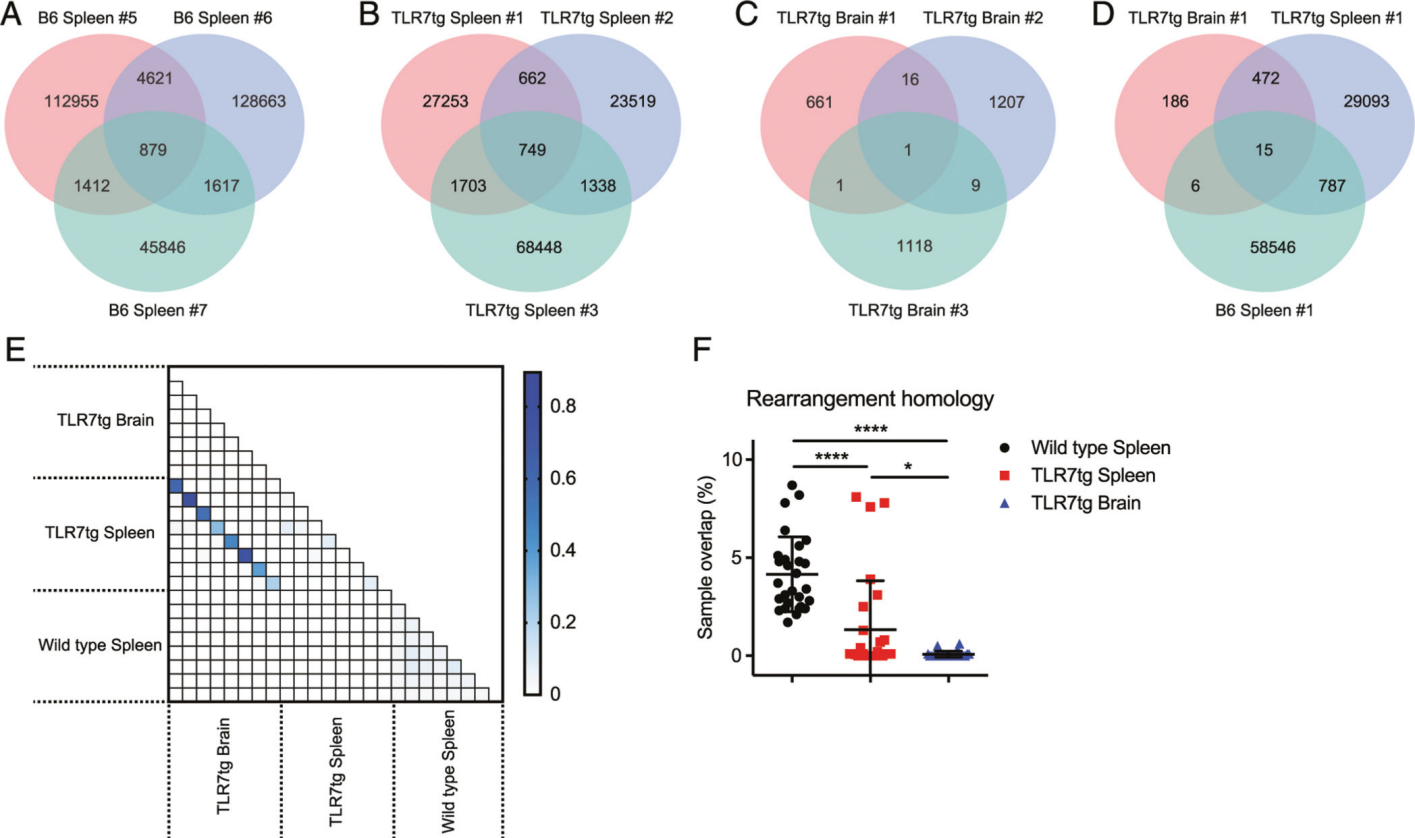
The CD8^+^ T cell repertoire is largely similar between the spleen and brain of the same lupus-prone animal. (**A–D**) Venn-diagram analysis of CDR3 nucleotide sequence homology from three wild type B6 spleens (A), three TLR7tg spleens (B), three TLR7tg brains (C), and across all three groups (D) comparing in-frame reads. (**E** and **F**) CDR3 nucleotide sequence overlap across all in-frame reads of samples (*n* = 8) from indicated groups calculated by Morisita Index (E) and summarized for each group (F). Error bars indicate mean + SD. *p ≤ 0.05, ****p ≤ 0.0001, by Student *t* test.

**FIGURE 6.**
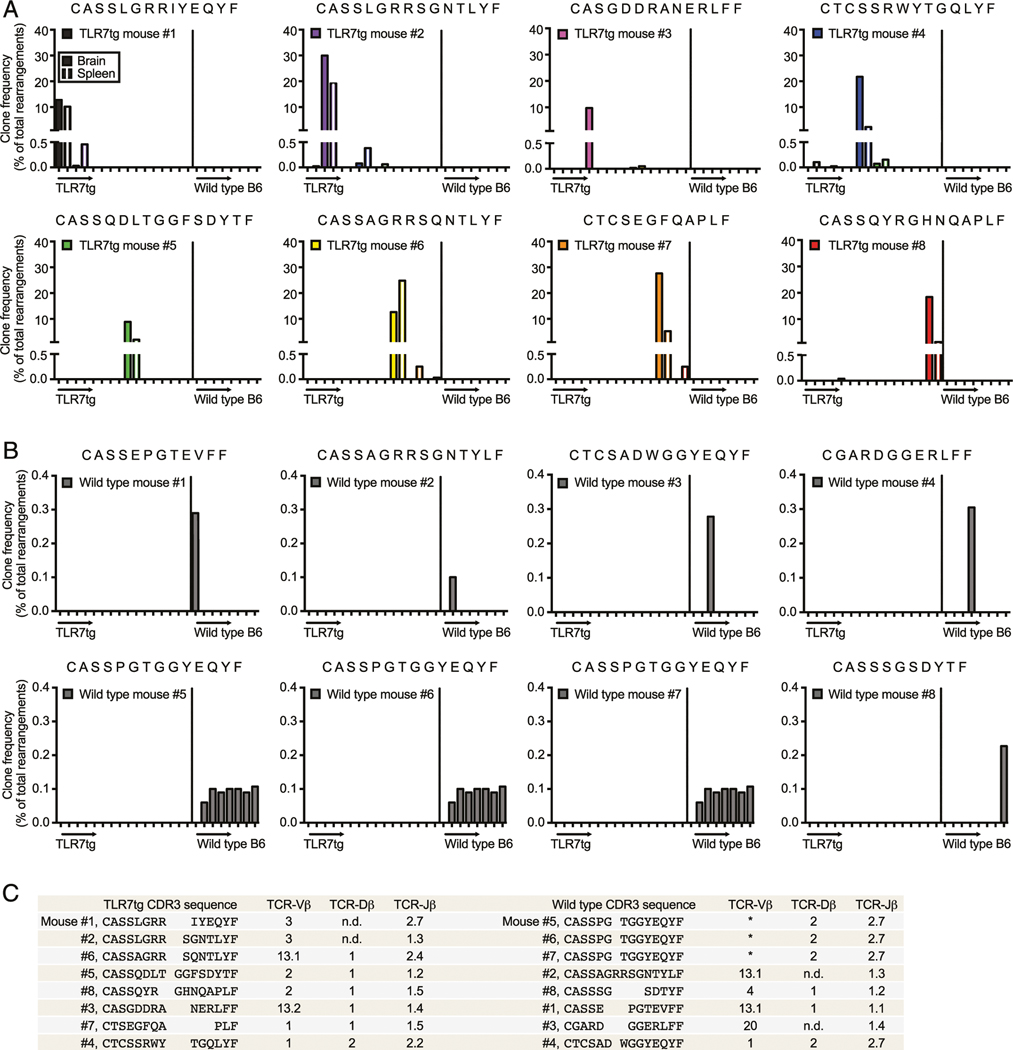
Regions of sequence homology across dominant clones in TLR7tg animals. (**A** and **B**) The CDR3 sequence of the dominant CD8^+^ T cell clone isolated from the brain of each TLR7tg (*n* = 8) (A) or spleen of each wild type B6 (*n* = 8) (B) mouse is shown. The frequency at which each of these top clones appears across all animals is presented. (**C**) CDR3 alignment along with V, D, and J gene usage for each top clone in TLR7tg or wild type B6 mice. An asterisk (*) indicates an instance in which multiple nucleotide sequences encoding different Vβ genes generated identical CDR3 amino acid sequences.

To better understand the substantial mouse-to-mouse variations in TLR7tg samples despite similar levels of oligoclonality, we analyzed absolute CDR3 nucleotide homology across all in-frame rearrangements from every animal tested between spleen and brain tissues of both genotypes (Fig. 5A–F). We identified a small number of shared identical CDR3 sequences between various wild type B6 splenic samples (Fig. 5A, 5E, 5F). Unexpectedly, a significant reduction in sharing across splenic TLR7tg mice was evident (Fig. 5B, 5E, 5F). We found additional reduction in such sharing across all brain-isolated populations (Fig. 5C, 5E, 5F), in some cases down to only a single shared sequence between two genetically identical animals. Within the same animal, we did find considerable instances of complete nucleotide homology in the CD8^+^ T cells between spleen and brain tissues (Fig. 5D, 5E), including sharing of the most abundant clone in the two tissues (Fig. 6A). These results indicate that lymphocyte expansion in a chronic-inflammatory autoimmune setting can give rise to an oligoclonal population of CD8^+^ T cells in which the same clones are likely to be found in both lymphoid and nonlymphoid tissues, but only within the same animal.

### Dominant clones across lupus-prone mice share regions of sequence homology but are distinct from the most common clones in healthy control animals

Remarkably, whereas the most abundant single clone in each wild type control sample was only between 0.1 and 0.4% of the entire population, in TLR7tg mice, this number reached between 10 and 30% for splenic samples (Fig. 6A). Similar percentages were found for brain-isolated CD8^+^ T cells from TLR7tg mice. The CDR3 amino acid sequences of the top rearrangements from wild type and TLR7tg mice were largely distinct (Fig. 6B), indicating that the CD8^+^ T cells selected by the environment in lupus-prone mice are different from the top clones in wild type controls.

Despite the strong oligoclonality we witnessed in the lupus-prone samples, complete nucleotide homology across various TLR7tg mice was rare (Fig. 5). A possible scenario is that similar but not identical CDR3 sequences might be selected by the same Ag within expanded T cell populations in these animals. We identified several highly similar dominant clones, particularly those from TLR7tg mice no. 1, 2, and 6, which contained up to 93% amino acid sequence homology while representing nearly one third of the CD8 repertoire in each animal (Fig. 6A, 6C). Interestingly, even though there was such a high degree of sequence homology in these clones, their VDJ gene usage was largely distinct. These results collectively indicate that clonal expansion in TLR7tg lupus-prone mice selects for particular dominant rearrangements that share regions of sequence homology despite not deriving from the same parent lymphocyte.

## DISCUSSION

The specificities and clonality of the lymphocytes thought to be involved in systemic lupus are not a clearly understood aspect of the disease, particularly as they concern CD8^+^ T cells. Previous studies found clonal expansion within the CD8^+^ T cell pool isolated from the kidneys of lupus patients (19), whereas an analysis of T cell Vβ gene usage and the Ag-determining CDR3 region revealed significant changes compared with healthy controls, indicative of more oligoclonal T cell populations (20, 31). The data in our current study support the notion of a narrowing TCR repertoire in lupus, as we find increased oligoclonality in the TLR7tg CD8^+^ T cell pool compared with wild type controls: a reduction of the total number of unique clones (Fig. 2), a diversion of CDR3 length away from expected Gaussian distribution (Fig. 3), and skewed VDJβ gene usage (Fig. 4). Importantly, our data suggest that strong selective pressures exist that shape the T cell repertoire, which extend beyond underlying genetics, as there was only partial sequence homology of dominant clones across various TLR7tg animals.

The level of clonality we observe in the TLR7tg mice, with a single clone comprising up to 30% of the entire T cell pool, is similar to what has been published in CD8-dependent models of viral infection such as lymphocytic choriomeningitis virus (32) and influenza (33). An important distinction between the TLR7tg lupus-prone mouse model and noted viral infection systems is the scope of available Ags to which the T cells can respond. Indeed, only three predominant CD8^+^ T cell clones have been described against the Armstrong strain of lymphocytic choriomeningitis virus (32). Importantly, although each of these clones contain unique VDJ genes, CDR3 lengths, and nucleotide sequences, they are consistently present across all infected animals. In our lupus model system, despite a similar degree of oligoclonality in the CD8^+^ T cell pool there is an unexpectedly broad and variable usage of VDJ genes, CDR3 lengths, and sequences across animals. The importance of this distinction remains unclear, although it is likely indicative of a heterogeneous response against a group of self-antigens. Monozygotic twins concordant for lupus begin life with virtually identical TCR repertoires, but their Vβ usage specifically in the CD8 pool diverges over time, suggesting that both genetics and a unique history of environmental exposure shape the T cell repertoire over time (34).

In a previous study, we showed that the CD8^+^ T cells in TLR7tg mice are activated cells that, compared with other lymphocytes in the animal, could traffic to and take up residence in the brain (7). Activated lymphocytes gain adhesive properties, allowing them to attach to blood vessel endothelium of tissues into which they will migrate (35). The most highly activated CD8^+^ T cells in TLR7tg mice upregulated many adhesion and trafficking markers (7), and so we posited that these cells would enter the brain and expand in response to some Ag-specific signals. Unexpectedly, our current data identified a markedly similar TCRβ repertoire between the peripheral and brain-resident CD8^+^ T cell pools in each respective animal (Figs. 5D, 5E, 6A). It is clear that of all the possible comparisons we could make across tissues and genotypes within a single animal, the strongest degree of sequence homology existed between cells within different tissues of the same animal. We conclude from these results that the pool of expanded, oligoclonal, mature lymphocytes we identified in both the spleen and brain of TLR7tg mice develop in response to the same selective pressures, although it remains unclear if the origin of such selection is from peripheral or brain Ags.

Nucleotide homology of the Ag-determining CDR3 region can provide some information about the CD8^+^ T cell repertoire. Although absolute homology across TLR7tg mice is rare, particularly in the brain, it is possible that the high degree of similarity identified across some of the top rearrangements is indicative of different clones arising with similar specificities, perhaps for some common self-antigen. This is possible even in cases when the different VDJ genes are used to make two unique rearrangements. For example, whereas a close relationship between the CDR3 specificity and the structure of the Ag receptor exists, and most of the T cells with the same MHC:peptide specificity were found to use the same αβ-chain combinations (36, 37), rare examples exist of various insulin-specific T cell clones and hybridomas using different Vα and Vβ genes (38, 39).

One interesting implication of our data relates to the notion that shrinking receptor diversity resulting from clonal expansion in individuals with chronic disease such as systemic lupus could promote what has been referred to as an “immune risk phenotype” (40) during senescence. Such a phenotype is predictive of mortality in aged individuals who are left more susceptible to constant immune insult from foreign pathogens. The narrowing of the lymphocyte repertoire, which is thought to impact CD8^+^ T cells more than CD4^+^ T cells, decreases immune defense particularly during senescence in both mice and humans (40–42). Because we see a narrowing lymphocyte repertoire in the context of autoimmune disease, it will be important in the future to use our lupus-prone mice and other such models to study the impact of chronic systemic disease on the ability of the organism to mount both effective primary and secondary immune responses to pathogens.

The Ag specificity of the TLR7tg CD8^+^ T cells we identified remains unknown, making it hard to speculate what might be driving the mouse-to-mouse differences despite similar overall clonality. Certainly, the frequency and sequence homology among the most abundant clones suggests a strong selective pressure indicative of some self-antigen–driven expansion of CD8^+^ T cells. However, how the top clones in the CD8^+^ T cell pool end up as part of the response in lupus-prone mice to begin with and why the repertoire is so variable between animals remains unclear and warrants further investigation.

## Abbreviation used in this article:

IMGT: ImMunoGeneTics information system
